# *OTOF* mutation analysis with massively parallel DNA sequencing in 2,265 Japanese sensorineural hearing loss patients

**DOI:** 10.1371/journal.pone.0215932

**Published:** 2019-05-16

**Authors:** Yoh-ichiro Iwasa, Shin-ya Nishio, Akiko Sugaya, Yuko Kataoka, Yukihiko Kanda, Mirei Taniguchi, Kyoko Nagai, Yasushi Naito, Tetsuo Ikezono, Rie Horie, Yuika Sakurai, Rina Matsuoka, Hidehiko Takeda, Satoko Abe, Chiharu Kihara, Takashi Ishino, Shin-ya Morita, Satoshi Iwasaki, Masahiro Takahashi, Tsukasa Ito, Yasuhiro Arai, Shin-ichi Usami

**Affiliations:** 1 Department of Otorhinolaryngology, Shinshu University School of Medicine, Matsumoto, Japan; 2 Department of Otolaryngology-Head and Neck Surgery, Okayama University Graduate School of Medicine, Dentistry and Pharmaceutical Sciences, Okayama, Japan; 3 Kanda ENT Clinic, Nagasaki Bell Hearing Center, Nagasaki, Japan; 4 Department of Otolaryngology-Head and Neck Surgery, Graduate School of Medicine, Kyoto University, Kyoto, Japan; 5 Department of Otolaryngology-Head and Neck Surgery, Gunma University Graduate School of Medicine, Maebashi, Japan; 6 Department of Otolaryngology, Kobe City Medical Center General Hospital, Kobe, Japan; 7 Department of Otorhinolaryngology, Saitama School of Medicine, Moroyama, Japan; 8 Shiga Medical Center for Children, Shiga, Japan; 9 Department of Otorhinolaryngology, Jikei University School of Medicine, Tokyo, Japan; 10 Department of Otorhinolaryngology, Juntendo University Faculty of Medicine, Tokyo, Japan; 11 Department of Otorhinolaryngology, Toranomon Hospital, Tokyo, Japan; 12 Department of Otolaryngology-Head and Neck Surgery, Nagasaki University Graduate School of Biomedical Sciences, Nagasaki, Japan; 13 Department of Otorhinolaryngology, Head and Neck Surgery, Hiroshima University Hospital, Hiroshima, Japan; 14 Department of Otolaryngology-Head and Neck Surgery, Faculty of Medicine and Graduate School of Medicine, Hokkaido University, Sapporo, Japan; 15 Department of Otorhinolaryngology, International University of Health and Welfare, Mita Hospital, Tokyo, Japan; 16 Department of Otolaryngology, Head and Neck Surgery, Yamagata University Faculty of Medicine, Yamagata, Japan; 17 Department of Otorhinolaryngology-Head and Neck Surgery, Yokohama City University School of Medicine, Yokohama, Japan; NIDCR/NIH, UNITED STATES

## Abstract

The *OTOF* gene (Locus: DFNB9), encoding otoferlin, is reported to be one of the major causes of non-syndromic recessive sensorineural hearing loss, and is also reported to be the most common cause of non-syndromic recessive auditory neuropathy spectrum disorder (ANSD). In the present study, we performed *OTOF* mutation analysis using massively parallel DNA sequencing (MPS). The purpose of this study was to reveal the frequency and precise genetic and clinical background of *OTOF*-related hearing loss in a large hearing loss population. A total of 2,265 Japanese sensorineural hearing loss (SNHL) patients compatible with autosomal recessive inheritance (including sporadic cases) from 53 otorhinolaryngology departments nationwide participated in this study. The mutation analysis of 68 genes, including the *OTOF* gene, reported to cause non-syndromic hearing loss was performed using MPS. Thirty-nine out of the 2,265 patients (1.72%) carried homozygous or compound heterozygous mutations in the *OTOF* gene. It is assumed that the frequency of hearing loss associated with *OTOF* mutations is about 1.72% of autosomal recessive or sporadic SNHL cases. Hearing level information was available for 32 of 39 patients with biallelic *OTOF* mutations; 24 of them (75.0%) showed profound hearing loss, 7 (21.9%) showed severe hearing loss and 1 (3.1%) showed mild hearing loss. The hearing level of patients with biallelic *OTOF* mutations in this study was mostly severe to profound, which is consistent with the results of past reports. Eleven of the 39 patients with biallelic *OTOF* mutations had been diagnosed with ANSD. The genetic diagnosis of *OTOF* mutations has significant benefits in terms of clinical decision-making. Patients with *OTOF* mutations would be good candidates for cochlear implantation; therefore, the detection of *OTOF* mutations is quite beneficial for patients, especially for those with ANSD.

## Introduction

Hearing loss is one of the most frequent congenital sensory disorders, with one out of every 500 newborns having bilateral hearing loss[1]. It is reported that 50–60% of these cases show a genetic etiology, with 80% of them demonstrating autosomal recessive hearing loss[2]. The *OTOF* gene (Locus: DFNB9), encoding otoferlin, is reported to be one of the frequent causes of non-syndromic recessive sensorineural hearing loss. To date, more than 160 mutations in *OTOF* have been reported, and most of the patients with *OTOF* mutations have stable, prelingual and severe to profound hearing loss. *OTOF* is also known to be the most common cause of non-syndromic recessive auditory neuropathy spectrum disorder (ANSD)[3–5]. ANSD is a unique form of hearing loss characterized by the absence of or severe abnormalities in auditory brainstem response (ABR) and the presence of otoacoustic emissions (OAE). *OTOF* is mainly expressed in cochlear inner hair cells, and is necessary for synaptic exocytosis at the ribbon synapse[6]. While the function of the inner hair cells is impaired, that of the outer hair cells is preserved for the first one or two years; therefore, hearing loss due to *OTOF* gene mutation can also present as ANSD.

Recently, targeted exon sequencing of selected genes using massively parallel DNA sequencing (MPS) technology has been developed, enabling us to analyze massive amounts of data both relatively quickly and inexpensively improve the molecular diagnostic rate of hearing loss patients [7–9]. Although we previously reported the prevalence of hearing loss with *OTOF* mutations on the basis of Sanger sequencing[5], it is both time-consuming and costly to analyze a large number of patients by this method as the *OTOF* gene has a large number of exons. In this study, we conducted a genetic analysis of the *OTOF* gene in 2,265 Japanese hearing loss patients by MPS. The purpose of this study was to reveal the frequency and precise genetic and clinical background of *OTOF*-related hearing loss in a large hearing loss population.

## Subjects and methods

### Subjects

A total of 2,265 Japanese sensorineural hearing loss (SNHL) patients compatible with autosomal recessive inheritance (including sporadic cases) from 53 otorhinolaryngology departments nationwide participated in this study. Hearing loss was evaluated using pure-tone audiometry (PTA) classified by a pure-tone average over 500, 1000, 2000 and 4000 Hz in the better hearing ear. For children who could not undergo PTA, we used an average over 500, 1000, 2000 Hz for either auditory steady stem response (ASSR) or conditioned oriented reflex audiometry (COR), or the response threshold (dBnHL) from ABR. The severity of hearing loss was classified as follows: normal hearing, <25dB; mild hearing loss, 25-39dB; moderate hearing loss, 40-69dB; severe hearing loss, 70-89dB; and profound hearing loss, greater than 90dB. Written informed consent was obtained from all subjects (or from their next of kin, caretaker, or guardian on the behalf of minors/children) prior to enrollment in the project. All procedures were approved by the Shinshu University Ethical Committee and the ethical committees of the other participating institutions listed as follows: Hokkaido University, Sapporo Medical University, Akita University, Iwate Medical University, Tohoku University, Tohoku Rosai Hospital, Yamagata University, Fukushima Medical University, Jichi Medical University, Gunma University, Jyuntendo University, Yokohama City University, Tokai University, Mejiro University, National Rehabilitation Center, Nihon University School, Saitama Medical University, Tokyo Medical University, Jikei University, Abe ENT clinic, Toranomon Hospital, Kitasato University, Tokyo Medical Center Institute of Sensory Organs, International University Health and Welfare Mita Hospital, Jichi University Saitama Medical Center, Aichi Children’s Health Medical Center, Chubu Rosai Hospital, Mie Hospital, Kyoto University, Kyoto Prefectural University, Mie University, Shiga Medical Center for Children, Shiga Medical University, Osaka University, Kansai Medical University, Kobe University, Osaka Medical Center and Research Institute for Maternal and Children Health, Hyogo College of Medicine, Okayama University, Kobe City Medical Center General Hospital, Wakayama Medical University, Kouchi University, Hiroshima University, Hiroshima City Hiroshima Citizen Hospital, Yamaguchi University, Ehime University, Kyushu University, Fukuoka University, Kurume University, Nagasaki University, Kanda ENT Clinic, Miyazaki Medical College, Kagoshima University, Ryukyus University.

### Variant analysis

Amplicon libraries were prepared using an Ion AmpliSeq Custom Panel (Applied Biosystems, Life Technologies), according to the manufacturer’s instructions, for 68 genes including all the exons of the *OTOF* gene (NM_194248, NM_194323) reported to cause non-syndromic hearing loss (S1 Table). The detailed protocol was described elsewhere[10]. After preparation, the amplicon libraries were diluted to 20pM and equal amounts of 6 libraries for 6 patients were pooled for one sequence reaction.

Emulsion PCR and sequencing were performed according to the manufacturer’s instructions. The detailed protocol was described elsewhere[10]. MPS was performed with an Ion Torrent Personal Genome Machine (PGM) system using an Ion PGM 200 Sequencing Kit and an Ion 318 Chip (Life Technologies).

The sequence data were mapped against the human genome sequence (build GRCh37/hg19) with a Torrent Mapping Alignment Program. After sequence mapping, the DNA variant regions were piled up with Torrent Variant Caller plug-in software. After variant detection, their effects were analyzed using ANNOVAR software[11, 12]. The missense, nonsense, insertion/deletion and splicing variants were selected from among the identified variants. Variants were further selected as less than 1% of 1) the 1,000 genome database[13], 2) the 6,500 exome variants (http://evs.gs.washington.edu/EVS/), 3) the Human Genetic Variation Database (dataset for 1,208 Japanese exome variants)[14], 4) the 333 in-house Japanese normal hearing loss controls, and 5) 1,000 control data in the deafness variation database[15]. All the mutations found in this study were confirmed by Sanger sequencing using exon-specific custom primers.

To predict the pathogenicity of the missense variants, we used 12 functional prediction software programs including ANNOVAR (SIFT, Polyphen2 HVID, Polyphen2 HVAR, LRT, Mutation Taster, Mutation Assessor, FATHMM, Radial SVM, LR, GERP++, PhyloP, SiPhy 29-way log odds and CADD).

## Results

Hearing level of the participating 2,265 patients was diagnosed as follows: mild hearing loss, 215 patients; moderate hearing loss, 679 patients; severe hearing loss, 524 patients; profound hearing loss, 599 patients; and unknown, 248 patients. The mutations found in this study were categorized into pathogenic, likely pathogenic, benign, likely benign and variant of uncertain significance according to the ACMG (American College of Medical Genetics) standards and guidelines[16]. The mutations judged to be pathogenic variants and likely pathogenic variants are presented in Table 1. Ten mutations including 6 previously reported variants and 4 novel variants (p.R425X, p.Y474X, p.W717X, p.L1003fs, p.Y1064X, p.Q1072X, p.I1449fs, p.R1856Q, p.R1172Q, c.4960+2T>C) were categorized as pathogenic variants. Five mutations (p.P489S, p.H513R, p.R1583H, p.R1792C, p.R1792H) were categorized as likely pathogenic variants. The 5 likely pathogenic variants were thought to be likely pathogenic because 1) they were found with previously reported pathogenic variants in *trans* (in different alleles of the gene): PM3 (p.R1856Q or p.R1172Q), 2) they were not found in the control: PM2, 3) the prediction programs scores support their pathogenicity: PP3 and 4) co-segregation with family members with disease: PP1. The mutations judged to be likely benign and variants of uncertain significance are presented in S2 Table. Ten mutations (p.G36A, p.G123S, p.I622V, p.E643K, p.R652Q, p.R654Q, p.R818W, p.V1012A, p.R1249W, c.4023+1G>A) were categorized as likely benign variants because 1) the allele frequency was greater than expected for the disorder (p.R818W): BS1, 2) the prediction programs scores did not support their pathogenicity (p.G36A, p.G123S, p.I622V, p.E643K, p.R652Q, p.R654Q, p.R1249W): BP4, 3) the variant was found in a case with an alternate molecular basis for disease (p.R652Q is found with homozygote *CDH23* mutations; p.V1012A is found with compound heterozygote *GJB2* mutations; c.4023+1G>A is found with compound heterozygote *SLC26A4* mutations or mitochondrial 3243A>G mutations): BP5 and 4) a reputable source (deafness variation database[17]) reports these mutations as benign or likely benign variants (p.G36A, p.G123S, p.I622V, p.E643K, p.R652Q, p.R654Q, p.R818W, p.V1012A, p.R1249W, c.4023+1G>A): BP6.

**Table 1:** The pathogenic and likely pathogenic variants of OTOF identified in this study.

	Nucleotide Change	Amino acid Change	Occurrence in this work (chromosome)	Control (chromosome)	Functional Prediction						Reference
					SIFT	PP2	LRT	Mut Taster	Mut Assessor	CADD	
Pathogenic											
NM_194248	c.1273C>T	p.R425X	1/4530	0/666	−	−	D(1)	A(1)	−	38	Tang et al., 2017
NM_194248	c.1422T>A	p.Y474X	8/4530	0/666	−	−	D(1)	A(1)	−	35	Matsunaga et al., 2012
NM_194248	c.2151G>A	p.W717X	1/4530	0/666	−	−	D(1)	A(1)	−	40	Iwasa et al., 2013
NM_194248	c.3007_3008del	p.L1003fs	1/4530	0/666	−	−	−	−	−	−	This study
NM_194248	c.3192C>G	p.Y1064X	2/4530	0/666	−	−	D(1)	A(1)	−	38	Bae et al., 2013
NM_194248	c.3214C>T	p.Q1072X	2/4530	0/666	−	−	D(1)	A(1)	−	41	This study
NM_194248	c.4346_4347insGCAT	p.I1449fs	1/4530	0/666	−	−	−	−	−	−	This study
NM_194248	c.4960+2T>C	−	1/4530	0/666	−	−	−	D(1)	−	23.6	This study
NM_194248	c.5567G>A	p.R1856Q	4/4530	0/666	D(0.72)	P(0.60)	D(0.84)	D(0.81)	M(0.78)	26	Choi et al., 2009
NM_194323	c.3515G>A	p.R1172Q	63/4530	0/666	D(0.72)	D(0.81)	−	D(0.81)	−	19.42	Varga et al., 2003
Likely pathogenic											
NM_194248	c.1465C>T	p.P489S	1/4530	0/666	D(0.91)	D(0.92)	D(0.84)	D(0.81)	M(0.66)	27.6	This study
NM_194248	c.1538A>G	p.H513R	1/4530	0/666	D(0.91)	D(0.67)	D(0.84)	D(0.81)	M(0.75)	25.5	This study
NM_194248	c.4748G>A	p.R1583H	1/4530	0/666	D(0.91)	D(0.97)	D(0.84)	D(0.81)	H(0.93)	35	Iwasa et al., 2013
NM_194248	c.5374C>T	p.R1792C	1/4530	0/666	D(0.91)	P(0.85)	D(0.84)	D(0.81)	M(0.92)	34	This study
NM_194248	c.5375G>A	p.R1792H	1/4530	0/666	D(0.91)	D(0.81)	D(0.84)	D(0.59)	M(0.92)	34	Almontashiri et al., 2017

A, disease causing automatic (MutationTaster); D, disease causing (MutationTaster), deleterious (SIFT) or probably damaging (PolyPhen2); H, high (MutationAssessor); L, low (MutationAssessor); M, medium (MutationAssessor); P, possibly damaging (PolyPhen2)

All of the patients with biallelic *OTOF* mutations are shown in Table 2. Here, the possible causative mutations in 68 deafness genes analyzed by NGS is also indicated.

**Table 2:** Cases with biallelic *OTOF* mutations in this study.

Patient ID	Mutation 1		Mutation 2		Severity*	Other pathogenic or likely pathogenic variants identified in same case
	Nucleotide change	Amino acid change	Nucleotide change	Amino acid change		
2703	c.3515G>A	p.R1172Q	c.3515G>A	p.R1172Q	NA	None
4908**	c.3515G>A	p.R1172Q	c.3515G>A	p.R1172Q	profound	CDH23:NM_022124:c.[1167C>A];[4762C>T]:p.[Y389X];[R1588W]
5058	c.3515G>A	p.R1172Q	c.3515G>A	p.R1172Q	profound	None
5082	c.3515G>A	p.R1172Q	c.3515G>A	p.R1172Q	profound	None
JHLB0047	c.3515G>A	p.R1172Q	c.3515G>A	p.R1172Q	severe	None
JHLB2693	c.3515G>A	p.R1172Q	c.3515G>A	p.R1172Q	profound	CDH23:NM_022124:c.[4762C>T]; [=]:p.[R1588W]; [=]
HL2270	c.3515G>A	p.R1172Q	c.3515G>A	p.R1172Q	NA	None
JHLB3180	c.3515G>A	p.R1172Q	c.3515G>A	p.R1172Q	profound	None
HL2581	c.3515G>A	p.R1172Q	c.3515G>A	p.R1172Q	profound	None
JHLB0264	c.3515G>A	p.R1172Q	c.3515G>A	p.R1172Q	profound	SLC26A4:NM_000441:c.[757A>G]; [=]:p.[I253V]; [=]
JHLB1281	c.3515G>A	p.R1172Q	c.3515G>A	p.R1172Q	profound	None
JHLB0105	c.3515G>A	p.R1172Q	c.3515G>A	p.R1172Q	profound	None
JHLB3948	c.3515G>A	p.R1172Q	c.3515G>A	p.R1172Q	profound	None
JHLB4045	c.3515G>A	p.R1172Q	c.3515G>A	p.R1172Q	severe	CDH23:NM_022124:c.[4762C>T]; [=]:p.[R1588W]; [=]
HL3598	c.3515G>A	p.R1172Q	c.3515G>A	p.R1172Q	NA	SLC26A4:NM_000441:c.[1983C>A]; [=]:p.[D661E]; [=]
HL3904	c.3515G>A	p.R1172Q	c.3515G>A	p.R1172Q	NA	None
JHLB2799	c.3515G>A	p.R1172Q	c.5567G>A	p.R1856Q	profound	GJB2:NM_004004:c.[109G>A]; [=]:p.[V37I]; [=]
JHLB2868	c.3515G>A	p.R1172Q	c.1422T>A	p.Y474X	profound	GJB2:NM_004004:c.[293G>A]; [=]:p.[R98Q]; [=] MYO3A:NM_017433:c.[1669C>T]; [=]:p.[Q557X]; [=]
JHLB3087	c.3515G>A	p.R1172Q	c.1538A>G	p.H513R	severe	GJB2:NM_004004:c.[109G>A]; [=]:p.[V37I]; [=]
JHLB3509	c.3515G>A	p.R1172Q	c.3007_3008del	p.L1003fs	profound	None
4013	c.1422T>A	p.Y474X	c.5567G>A	p.R1856Q	profound	None
JHLB2430	c.5567G>A	p.R1856Q	c.1465C>T	p.P489S	profound	None
JHLB0001	c.3515G>A	p.R1172Q	c.3192C>G	p.Y1064X	profound	COL11A2:NM_080680:c.[1119+1G>A]; [=]
2529	c.3515G>A	p.R1172Q	c.3192C>G	p.Y1064X	profound	None
2958	c.3515G>A	p.R1172Q	c.2151G>A	p.W717X	severe	None
JHLB0098	c.3515G>A	p.R1172Q	c.1422T>A	p.Y474X	profound	None
HL0188	c.3515G>A	p.R1172Q	c.5374C>T	p.R1792C	NA	None
JHLB0892	c.3515G>A	p.R1172Q	c.1422T>A	p.Y474X	profound	None
JHLB2465	c.3515G>A	p.R1172Q	c.4960+2T>C	−	profound	None
JHLB1672	c.3515G>A	p.R1172Q	c.5375G>A	p.R1792H	profound	None
JHLB2300	c.3515G>A	p.R1172Q	c.1422T>A	p.Y474X	severe	None
JHLB1897	c.3515G>A	p.R1172Q	c.4346_4347insGCAT	p.I1449fs	profound	None
JHLB2536	c.3515G>A	p.R1172Q	c.3214C>T	p.Q1072X	NA	None
JHLB2576	c.3515G>A	p.R1172Q	c.3214C>T	p.Q1072X	profound	None
3098	c.3515G>A	p.R1172Q	c.4748G>A	p.R1583H	severe	None
JHLB1226	c.3205T>G	p.F1069V	c.5405C>T	p.A1802V	severe	None
JHLB2789	c.650A>G	p.D217G	c.5405C>T	p.A1802V	mild	None
JHLB2951	c.1780G>A	p.E594K	c.740G>A	p.S247N	profound	None
JHLB2370	c.3515G>A	p.R1172Q	c.1194T>A	p.D398E	NA	None

*average 500, 1000, 2000 and 4000Hz in the better hearing ear: 25-39dB: mild, 40-69dB: moderate, 70-89dB: severe, >90dB: profound

**This patient also carried compound heterozygous *CDH23* mutations. However, the clinical phenotype of this patient was congenital profound hearing loss and presumably caused by *OTOF* mutations. (The typical *CDH23* associated hearing loss involving the high frequency portion and residual hearing are usually observed in lower frequencies.)

Thirty-nine of the 2,265 patients (1.72%) carried homozygous or compound heterozygous mutations in the *OTOF* gene. Hearing level information was available for 32 of the 39 patients with biallelic *OTOF* mutations; 24 of them (75.0%) had profound hearing loss, 7 (21.9%) had severe hearing loss and 1 (3.1%) had mild hearing loss.

Only 11 of the 39 patients with biallelic *OTOF* mutations had been diagnosed with ANSD.

Clinical information regarding vertigo was available for 32 of the 39 patients with biallelic *OTOF* mutations, with 31 of them (96.9%) not experiencing any episodes of vertigo.

## Discussion

In this study, 39 (1.72%) of 2,265 SNHL patients compatible with autosomal recessive (including sporadic cases) inheritance carried homozygous or compound heterozygous mutations in the *OTOF* gene. Two patients with heterozygous mutations (p.Y474X and p.R1172Q) showed an ANSD phenotype, and it is strongly suspected that they had *OTOF* related deafness. Possible explanations for these heterozygous cases are 1) the co-existence of copy number variations, 2) the existence of a second mutation in the exonic region that could not be covered in this study or in regulatory region of *OTOF*, which was not explored, 3) the contribution to hearing loss of an additional modulatory gene, and 4) the existence of a mutation in another gene (*DIAPH3*, *AIFM1*, *ATP1A3* and mitochondrial 12SrRNA) which causes non-syndromic ANSD not examined in this study [18], so that the patients were just coincidental carriers of the *OTOF* mutations.

*DFNB59* gene (also called as *PJVK* gene), reported to be a cause of non-syndromic ANSD [19], was also included in this study and no mutation was found in these two patients. We also performed copy number variation analysis for 68 genes (including *OTOF* and *DFNB59*) but did not identified any copy number variations in these two patients. Therefore, it is assumed that the frequency of hearing loss patients with *OTOF* mutations is at least 1.72% among autosomal recessive or sporadic SNHL cases. It was previously reported that *OTOF* mutations accounted for 1.4–8.3% of non-syndromic hearing loss patients: 2.3% (13/557) in Pakistani[20], 3.2% (23/708) in Spanish[21], 8.3% (1/12) in Turkish[22], 2.6% (1/38) in Iranian[23] and 1.4% (1/73) in Chinese[24] populations. In this study, we analyzed 2,265 SNHL patients by MPS, which is the largest population analyzed to date. We had analyzed 160 SNHL patients by Sanger sequencing and reported that *OTOF* mutations accounted for 3.2–7.3% of recessive severe to profound SNHL[5]. This frequency is higher than that observed in this study. The main reason for this difference is thought to be that the subjects in this study included mild to moderate hearing loss cases. Our study included 1,123 patients with severe-profound hearing loss, and 30 (2.67%) of these patients had biallelic *OTOF* mutations, a rate which is comparable with that of our previous report.

The hearing levels in patients with biallelic *OTOF* mutations in this study were mostly severe to profound: 75.0% (24/32) had profound hearing loss, and 21.9% (7/32) had severe hearing loss. The commonly observed phenotype in patients with *OTOF* mutations is non-progressive, congenital and severe to profound hearing loss. This is consistent with the results of this study. Genotype-phenotype correlations of *OTOF* have been discussed in past reports [3, 18, 23]. Patients with truncating mutations (nonsense and frameshift) or splice-site mutations basically show severe to profound hearing loss. Concerning non-truncating mutations (missense mutation and in-frame deletion), hearing level varies depending on each mutation or co-existing mutation; therefore some of them could show mild to moderate hearing loss [18, 25]. In this study, only 1 patient (3.1%) had mild hearing loss; however, both mutations carried by the patient were variants of uncertain significance (p.D217G and p.A1802V), and it is unclear whether the true etiology of the hearing loss in this patient is due to mutations in the *OTOF* gene. Rare cases of temperature-sensitive ANSD, a particular form of ANSD, have been reported in some populations[3, 24]; however, no temperature-sensitive ANSD was observed in this study.

As shown in Table 2, p.R1172Q was frequently identified in the patients participating in this study. Sixteen of 39 patients (41.0%) with biallelic *OTOF* mutations had homozygous p.R1172Q mutations. Eighteen of 39 patients (46.2%) had p.R1172Q with another mutation in compound heterozygosity. In summary, 34 of 39 patients (87.2%) had at least one p.R1172Q mutation. p.R1172Q has been proven to be a founder mutation[3], and as it was quite frequently detected in this study we believe it to be an important mutation in Japanese SNHL patients. p.Y474X (12.8%: 5/39), p.R1856Q (7.7%: 3/39), p.Q1072X (5.1%: 2/39) and p.Y1064X (5.1%: 2/39) were also detected in two or more patients. Populations among the various races have different mutation spectra and recurrent mutations. p.Q829X is quite frequently detected in Spanish[26], c.2905_2923delinsCTCCGAGCGGCA in Argentinean[21], p.V1778F in Ashkenazi Jewish[27], p.E57X and p.R1792H in Saudi Arabian[28] and p.E1700Q in Taiwanese[25] populations. Each recurrent mutation among these populations means that *OTOF*-related hearing loss is a major etiology of hearing loss in the respective country, not only in Japan.

The significance of the genetic diagnosis of *OTOF* mutations lies in its benefits for clinical decision-making. *OTOF* mutations represent one of the etiologies of ANSD. ANSD is heterogeneous disorder, and the outcomes of cochlear implantation for patients with ANSD vary[29, 30]. Cochlear implantation has been reported to be effective for the patients with *OTOF* mutations as the *OTOF* gene mutations result in disruption of the synaptic exocytosis of inner hair cells, and the auditory nerves and spiral ganglions are preserved in patients with these mutations[31–33]. It is reported that outcome of cochlear implantation is predictable to some extent for various gene mutations[34]. Patients with *OTOF* mutations are predicted to show good outcomes; therefore, the detection of *OTOF* mutations is quite beneficial for the patients, especially for those with ANSD.

In this study, we also investigated whether the patients with biallelic *OTOF* mutations experience episodes of vertigo, and found that 31 of 32 patients (96.9%) with biallelic *OTOF* mutations had no such episodes. Although the *OTOF* gene is also expressed in vestibular hair cells, otoferlin-deficient mice show no evidence of apparent vestibular dysfunction based on gross evaluation by vestibular testing [6]. To date there have been no reports of a relationship between *OTOF* mutations and episodes of vertigo, and our results also showed that the clinical symptoms of vertigo are rarely observed in *OTOF*-related hearing loss patients.

In Japan, genetic testing for patients with SNHL using the Invader assay to screen for 46 mutations in 13 deafness genes was approved by the Ministry of Health, Labour and Welfare for inclusion in social health insurance coverage in 2012. Furthermore, the genetic testing was expanded in 2015 to allow screening for 154 mutations in 19 deafness genes using targeted genomic enrichment with MPS combined with the Invader assay [35]. We previously analyzed 717 hearing loss patients and achieved a 30% (212/717) diagnostic rate. *OTOF* mutations were also included among those 154 mutations and were identified in some cases. The pathogenic variants identified in this study will be added to this social health insurance-based genetic testing and further improvement in the diagnostic rate is expected.

## Supporting information

S1 Table 68 deafness-causative genes. (XLSX)

S2 Table The likely benign variants and variants of uncertain significance identified in this study. (XLSX)

S1 Fig The chromatograms of pathogenic and likely pathogenic variants identified in this study. The chromatograms of each variant (Upper row: variant, lower row: control).
(TIF)
