# Supplementary figures and images for: OTOF mutation analysis with massively parallel DNA sequencing in 2,265 Japanese sensorineural hearing loss patients
Source: PLoS One. 2019 May 16;14(5):e0215932. doi: 10.1371/journal.pone.0215932 (PMC6522017; doi:10.1371/journal.pone.0215932)

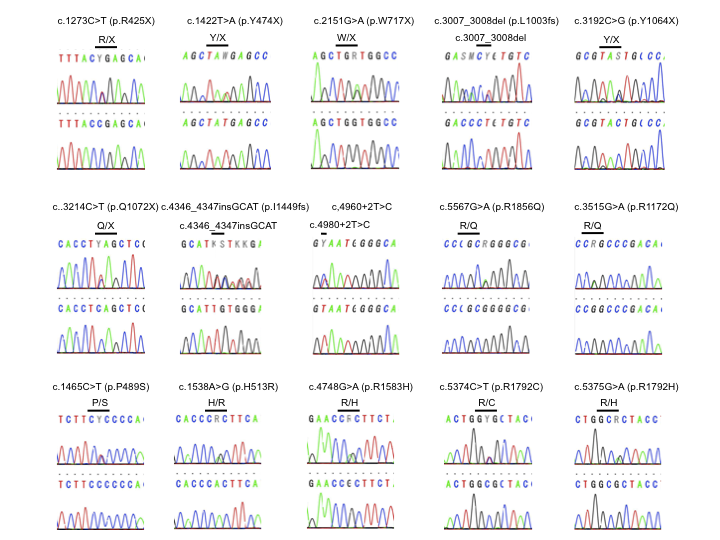

Supplement: S1 Fig — The chromatograms of each variant (Upper row: variant, lower row: control). (TIF) [file pone.0215932.s003.tif]
